# Gas Phase Hydrogenation of Furaldehydes via Coupling with Alcohol Dehydrogenation over Ceria Supported Au-Cu

**DOI:** 10.3390/molecules23112905

**Published:** 2018-11-07

**Authors:** Chiara Pischetola, Laura Collado, Mark A. Keane, Fernando Cárdenas-Lizana

**Affiliations:** Chemical Engineering, School of Engineering and Physical Sciences, Heriot Watt University, Edinburgh EH14 4AS, Scotland, UK; cp44@hw.ac.uk (C.P.); laura.lcb84@gmail.com (L.C.); M.A.Keane@hw.ac.uk (M.A.K.)

**Keywords:** HMF, coupling hydrogenation/dehydrogenation, *m-*furaldehydes, alcohols, Au/CeO_2_, Au-Cu/CeO_2_

## Abstract

We have investigated the synthesis and application of Au-Cu/CeO_2_ (Cu: Au = 2) in the continuous gas phase (*P =* 1 atm; *T* = 498 K) coupled hydrogenation of 5-hydroxymethyl-2-furaldehyde (HMF) with 2-butanol dehydrogenation. STEM-EDX analysis revealed a close surface proximity of both metals in Au-Cu/CeO_2_ *post*-TPR. XPS measurements suggest (support → metal) charge transfer to form Au*^δ^*^−^ and strong metal-support interactions to generate Cu^0^ and Cu^+^. Au-Cu/CeO_2_ promoted the sole formation of 2,5-dihydroxymethylfuran (DHMF) and 2-butanone in the HMF/2-butanol coupling with full hydrogen utilisation. Under the same reaction conditions, Au/CeO_2_ was fully selective to DHMF in standard HMF hydrogenation (using an external hydrogen supply), but delivered a lower production rate and utilised less than 0.2% of the hydrogen supplied. Exclusive -C=O hydrogenation and -OH dehydrogenation is also demonstrated for the coupling of a series of *m-*substituted (-CH_3_, -CH_2_CH_3_, -CH_2_OH, -CF_3_, -N(CH_3_)_2_, -H) furaldehydes with alcohol (1-propanol, 1-butanol, 2-propanol, 2-butanol, cyclohexanol) dehydrogenation over Au-Cu/CeO_2_, consistent with a nucleophilic mechanism. In each case, we observed a greater hydrogenation rate and hydrogen utilisation efficiency with a 3–15 times lower E-factor in the coupling process relative to standard hydrogenation. Our results demonstrate the feasibility of using hydrogen generated in situ through alcohol dehydrogenation for the selective hydrogenation of *m-*furaldehydes with important industrial applications.

## 1. Introduction

**Figure 1:**
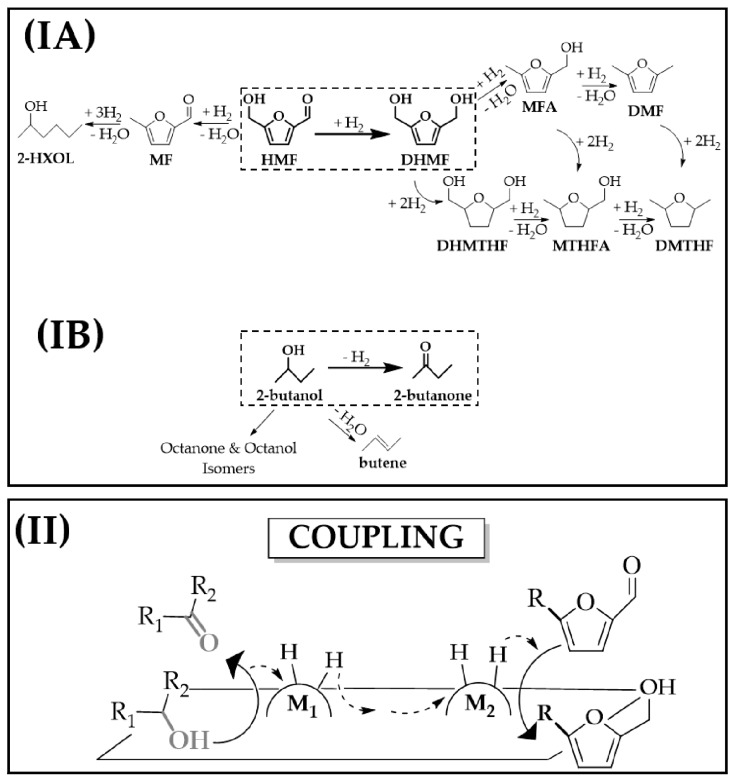
(**I**) Reaction scheme for (**A**) HMF hydrogenation to the target DHMF (bold arrow, dashed frame) and undesired by-products from hydrogenolysis (MF, MFA, DMF), ring reduction (DHMTHF, MTHFA, DMTHF) and ring opening (2-HXOL) and (**B**) 2-butanol dehydrogenation to the target 2-butanone (bold arrow, dashed frame) and undesired by-products from dehydration (butene) and dimerisation (octanone and/or octanol isomers). (**II**) Schematic representation of the coupling system. *Note: HMF = 5-hydroxymethyl-2-furaldehyde, MF = 5-methyl-2-furaldehyde, 2-HXOL = 2-hexanol, DHMF = 2,5-di-(hydroxymethyl)-furan, MFA = 5-methyl furfuryl alcohol, DMF = 2,5-dimethylfuran, DHMTHF = 2,5-di-(hydroxymethyl)-tetrahrydrofuran, MTHFA = 5-methyltetrahydro furfuryl alcohol, DMTHF = 2,5-dimethyltetrahydrofuran.*

5-hydroxymethyl-2-furaldehyde (HMF) has emerged as a promising biomass-derived platform chemical [1] that can be readily obtained from C_6_ carbohydrates (e.g., fructose, glucose) by dehydration in the presence of acid catalysts (e.g., metal halides, acid metal oxides) [2,3]. 2,5-Dihydroxymethylfuran (DHMF), a reduction product of HMF, is used as a precursor in the manufacture of polymer plastics (e.g., polyurethane foams, 16M tons per year [4]) resins and artificial fibres [5,6]. Conventional HMF→DHMF reduction involves standard catalytic hydrogenation in batch liquid phase [7] that suffers from safety and environmental issues due to the requirement for operating at high pressures (typically 5–100 bar) [7] under conditions with high excess of hydrogen in order to maximise H_2_ solubility and product yield. Hydrogen production requires *non-*renewable fossil fuel-based technologies such as steam and auto-thermal reforming [8]. Reaction selectivity is also challenging, as HMF hydrogen treatment can generate several products as shown in Figure 1(IA) [9,10,11] with Ru-catalysts delivering high combined activity/selectivity [7]. There is now a pressing demand for a more sustainable system that satisfies green chemistry principles to promote selective DHMF formation (i.e., #2, 7, 9 and 12 [12]). Several metrics have emerged to quantitatively assess green performance, most notably the environmental factor (E-factor, kg_waste_ kg_product_^−1^) [13]. E-factor values within the 5–100 range are characteristic of catalytic processes in the manufacture of pharmaceuticals and fine chemicals industries [13].

Hydrogenation can be effectively carried out in the absence of an external hydrogen supply through an alternative coupled process in which hydrogen generated in situ via (*non*-oxidative) dehydrogenation of alcohols (Figure 1(IB)) is utilised in a hydrogenation process (Figure 1(II)). This coupling strategy offers a series of advantages relative to stand-alone hydrogenation and dehydrogenation reactions including: (i) one-pot simultaneous production of two valuable chemicals (i.e., improved atom economy), (ii) good thermal efficiency (i.e. heat transfer from exothermic hydrogenation to endothermic dehydrogenation), (iii) enhanced yields (i.e. consumption in the hydrogenation step displaces the equilibrium of dehydrogenation increasing hydrogen production) and (iv) improved process safety (i.e. no need for pressurised H_2_) [14,15]. Despite the multiple advantages, it is a challenging system, with only a limited number of published studies on coupled heterogeneous catalytic systems. This is likely due to several factors, including: (i) a requirement for two active sites for dehydrogenation and hydrogenation (M_1_ and M_2_ in Figure 1(II)) that must be separated but in close proximity to facilitate hydrogen transport and utilisation, (ii) that sufficient hydrogen must be generated in the dehydrogenation step for the hydrogenation reaction and (iii) the possibility of cross reaction between reactants and/or products. It is nonetheless worth mentioning the promising results reported in the coupling of 1,4-butanediol [16] and *n*-butanol [17] dehydrogenation with maleic anhydride hydrogenation, the coupled of cyclohexanol/furfural [15,18,19,20] and 1,4-butanediol/furfural conversion [20,21,22,23]. Taking HMF → DHMF, the feasibility of coupling HMF with 1,4-butanediol over Cu-Al [24] and 2-butanol using zirconium hydroxides [25] has been explored only in batch liquid systems where the requirement of high pressure (16 bar) or low selectivity to target DHMF (*S*_DHMF_ ≤ 90%) are decided drawbacks. We were unable to find any study in the open literature dealing with DHMF production through a coupling process with HMF in continuous gas-phase operation.

We have recently showed the feasibility of coupling 2-butanol with furfural over a physical mixture of Au/CeO_2_ + Cu/CeO_2_ [26]. Minimising the physical separation between Au and Cu in bimetallic catalysts should facilitate hydrogen transfer/utilisation. In this work we evaluate the continuous gas phase dehydrogenation of alcohols (to ketones/aldehydes) coupled with hydrogenation of HMF to DHMF over Au-Cu supported on (non-toxic, [27]) CeO_2_. The catalytic action of Au-Cu/CeO_2_ to promote -C=O group reduction for a series of *m*-substituted furaldehydes has been investigated to understand reaction mechanism and establish the potential of the bimetallic catalyst. The benefits of the coupled process are examined by comparison with (conventional) stand-alone hydrogenation using an external hydrogen supply over Au/CeO_2_ as a benchmark and employing the E-factor as a measure of environmental performance.

## 2. Results and Discussion

### 2.1. Catalytic Conversion of 5-Hydroxymethyl-2-Furaldehyde (HMF): Coupling Dehydrogenation-Hydrogenation *vs.* Conventional Stand-Alone Hydrogenation Using an External H_2_ Supply

In the gas-phase coupled 2-butanol dehydrogenation with HMF hydrogenation, Au-Cu/CeO_2_ was 100% selective towards the production of targets 2-butanone and DHMF (Figure 1(II)). These two products can be readily separated by distillation due to the (195 K) difference in boiling points [28]. The formation of DHMF demonstrates utilisation of hydrogen generated in the dehydrogenation step for the continuous conversion of HMF. We could not find any report on DHMF production through alcohol/HMF coupling dehydrogenation/hydrogenation in continuous gas phase operation. It is, nonetheless, worth mentioning the recent work of Hu et al. [25], who investigated the coupled HMF/2-butanol process in batch liquid phase over magnetic zirconium hydroxides although they reported the undesired formation of 5-methyl-2-furaldehyde (MF) and 5-methylfurfuryl alcohol (MFA) with selectivity towards the target DHMF ≤ 90%. Exclusive formation of DHMF has proved difficult to fully circumvent also in standard hydrogenation, with unwanted over-hydrogenation (to DHMTHF, Figure 1(IA)) and/or hydrogenolysis (to MFA and DMF) reported in the liquid phase hydrogen treatment of HMF over supported Ni [10], Pd [11] and Ru [9] catalysts. In addition to DHMF selectivity, hydrogen utilisation efficiency is a key parameter that must be optimised to guarantee process sustainability. Full hydrogen utilisation was achieved in the coupled system with all the amount generated via 2-butanol dehydrogenation being utilised in HMF hydrogenation, i.e., H_2_/HMF = 1. We recorded no conversion in the stand-alone dehydrogenation of 2-butanol (in N_2_) over Au/CeO_2_ while under similar reaction conditions (*P* = 1 atm; *T* = 498 K) Cu/SiO_2_ delivers negligible activity in the gas phase hydrogenation of furfural [29]. This suggests that Cu and Au are the active sites for 2-butanol dehydrogenation and HMF hydrogenation, respectively. Alcohol dehydrogenation over supported copper [30] proceeds through a two-step mechanism that involves sequential H abstraction from O-H bond and α-carbon [26,31]. HMF adsorption can proceed via -C=O bond π-back donation on Au*^δ^*^−^ [32] or carbonyl O coordination with Au*^δ^*^+^ sites [33]. Spillover hydrogen species migrate from Cu → Au across the CeO_2_ surface [34] with auto-transfer in the catalytic conversion of HMF → DHMF.

We compared the catalytic response in the coupled vs. stand-alone process under the same reaction conditions of stoichiometric hydrogen supply (i.e. H_2_/HMF = 1). No conversion was detected over Au/CeO_2_ or Au-Cu/CeO_2_ in standard HMF hydrogenation using an external supply of H_2_. This lack of activity can be linked to the low capacity of Au for H_2_ chemisorption/activation [35,36], rate-limiting step in stand-alone hydrogenation [37]. Atomic hydrogen generated in situ in the coupled process during 2-butanol dehydrogenation is active for hydrogenation [38] and participates in HMF transformation, circumventing the issue of H_2_ activation by gold. An increase in hydrogen supply (H_2_/HMF = 80) in stand-alone hydrogenation enhanced the available surface H_2_ where both catalysts promote exclusive DHMF formation at similar production rates, but with less than 0.2% of the hydrogen supplied being utilised. This result demonstrates a requirement to work under conditions of hydrogen excess in the stand-alone process. Moreover, same activity/selectivity response over Au/CeO_2_ and Au-Cu/CeO_2_ further proves that the presence of Cu has no effect on the hydrogenation step which is governed by the gold component. We observed a 3.5-fold greater DHMF production rate in the coupling process relative to stand-alone hydrogenation over Au-Cu/CeO_2_. This demonstrates that the production of activated hydrogen over Cu by in situ alcohol dehydrogenation is more efficient than chemisorption of molecular H_2_ on Au via standard hydrogenation. Coupled reaction over Au-Cu/CeO_2_ was accompanied by lower E-factor (56) relative to standard hydrogenation in gas phase over Au/CeO_2_ (260) and batch mode using Ru catalysts (~100) [7]. Our results prove the benefits of the coupled process compared to standard hydrogenation for the transformation of HMF in terms of greater catalytic activity and full hydrogen utilisation efficiency with exclusive formation of target DHMF.

### 2.2. Catalyst Characterisation: Au-Cu/CeO_2_ and Au/CeO_2_

**Figure 2:**
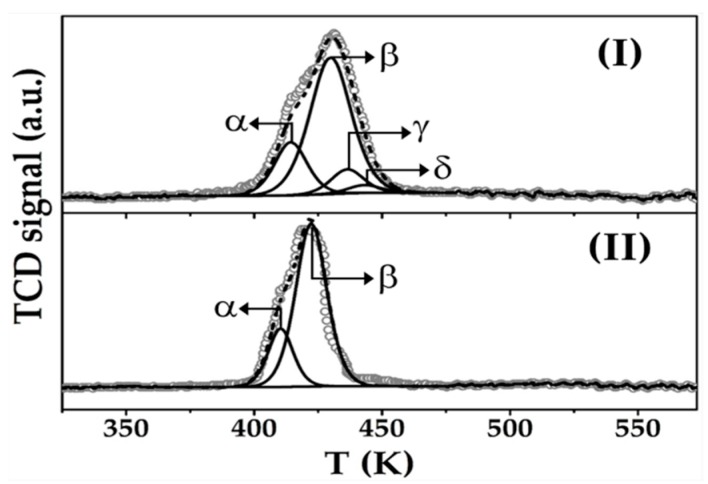
H_2_-Temperature programmed reduction (TPR) profiles for (**I**) Au-Cu/CeO_2_ and **(II)** Au/CeO_2_. *Note:* Raw data is shown as open symbols (**⚬**) while curve fitted and envelope is represented by solid and dashed lines, respectively.

The critical properties of Au-Cu/CeO_2_ and Au/CeO_2_ are presented in Table 1. Both catalysts exhibit a similar TPR profile (Figure 2) characterised by a broad positive (hydrogen consumption) peak centred at 427 ± 5 K that is within the reported temperature range (408–550 K) for the reduction of Au^3+^ → Au^0^ and transformation of Cu^2+^ to zero valent Cu [39,40]. Total H_2_ consumption exceeded (by up to a factor of 4) the requirement for complete reduction of the metal precursor(s), indicative of partial support reduction at the metal-CeO_2_ interface by spillover hydrogen [41]. Both catalysts present peaks with *T*_max_ at 412 ± 2 K (α) and 426 ± 3 K (β) characteristic of Au^3+^ → Au^0^ and Ce^4+^ → Ce^3+^, respectively [42,43]. The similar hydrogen consumption for α (67 ± 2 μmol g^−1^) and β (233 ± 5 μmol g^−1^) in both catalysts suggests same nature of Au and equivalent degree of CeO_2_ support reduction by spillover hydrogen promoted by gold. Limited capacity of Cu for H_2_ activation can account for the similar degree of CeO_2_ reduction and same catalytic response in stand-alone hydrogenation over Au-Cu/CeO_2_ and Au/CeO_2_. In addition, Au-Cu/CeO_2_ shows peaks characteristic of Cu^2+^ → Cu^+^ (γ centred at 437 K) and Cu^+^ → Cu^0^ (δ at 443 K) indicative of stepwise Cu reduction [44,45], where hydrogen consumption is consistent with a Cu^+^:Cu^0^ molar ratio = 3:1. The presence of cationic copper following TPR to 573 K can be linked to a stabilisation of Cu^+^ due to strong metal support interactions at Ce^3+^ sites [46,47]. Liu et al. [48], studying methanol steam reforming, showed (by XPS) the presence of Cu^+^ after reduction at *T* ≥ 573 K. 

**Figure 3:**
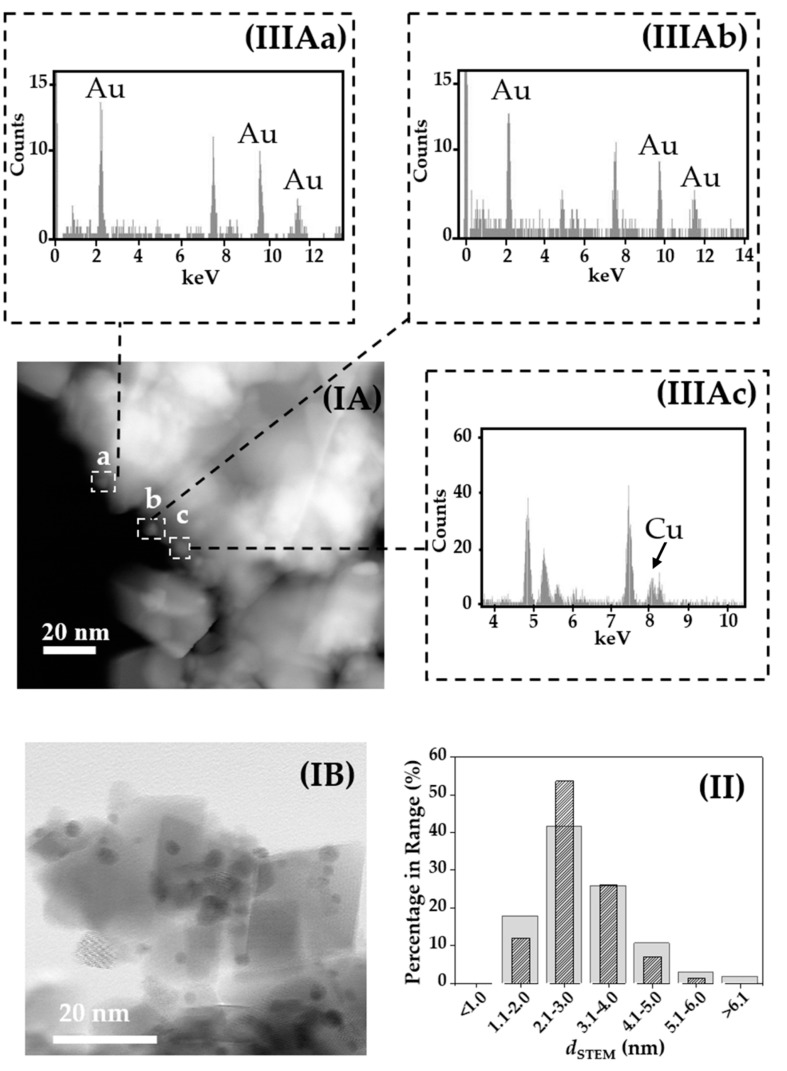
(**I**) Representative STEM images with (**II**) associated metal size distribution histogram for (**A**) Au-Cu/CeO_2_ (solid bars) and (**B**) Au/CeO_2_ (hatched bars). *Note*: STEM-EDX analyses of (**a–c**) isolated metal particles in (**IA**) are shown in (**IIIAa–IIIAc**).

Scanning transmission electron microscopy (STEM) measurements (Figure 3(I-II)) revealed a similar metal dispersion and mean size for both catalysts characterised by pseudo-spherical nano particles with an associated surface area weighted mean diameter of 3.5 ± 0.5 nm. The surface composition of the Au-Cu/CeO_2_ catalyst was probed by STEM/EDX elemental analysis. A detailed elemental analysis over small areas was carried out and the EDX spectra of three isolated metal particles (IIIAa–IIIAc) in Au-Cu/CeO_2_ are shown in Figure 3. The EDX spectra exhibit peaks due to nickel in the grid (7.5 and 8.3 keV) [49] and the CeO_2_ support (4.9–5.8 keV) [50]. The signals at 2.2, 9.7 and 11.5 keV in the EDX of particles a–b are characteristic of Au [50]. In contrast, particle c (IIIAc) shows only a peak at 8 keV consistent with the presence of Cu [50]. The detailed EDX analysis over single metal nanoparticles reveals a close proximity of Au and Cu on the surface. In the case of the Au-Cu system, the miscibility gap is such that bulk alloy formation is possible at *T* ≥ 490 K [51]. There is a dearth of literature dealing with Au-Cu systems; however, metal segregation for Au-Cu/CeO_2_ catalysts *post*-thermal treatment at *T* ≤ 573 has been proven theoretically [52] and experimentally [53] and attributed to strong interactions at the metal-CeO_2_ interphase. Zhang et al. [52] concluded that CeO_2_ support induces segregation for the Au-Cu system based on the CeO_2_-induced preferential segregation energy (*E*_seg,CeO2-Au_), a parameter indicating the strength of the Cu-CeO_2_ vs. Cu-Au bond energy. Ta and co-workers [54] showed by atomic resolution environmental transmission electron microscopy that gold nanoparticles of 2–4 nm strongly anchored onto CeO_2_ and did not sinter after reduction at *T* ≤ 573 K.

**Figure 4:**
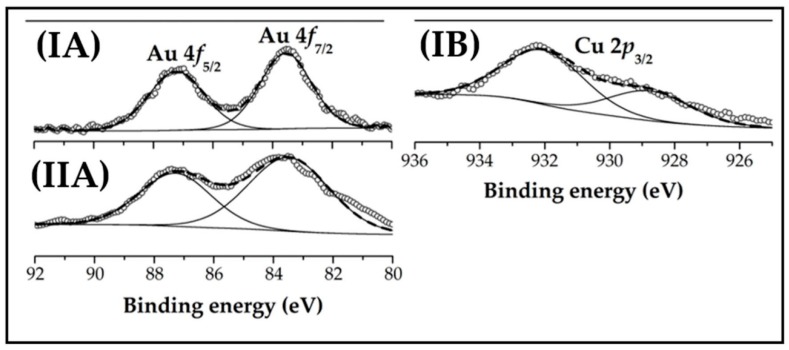
XPS spectra over the (**A**) Au 4*f* and (**B**) Cu 2*p*_3/2_ regions for (**I**) Au-Cu/CeO_2_ and (**II**) Au/CeO_2_. *Note:* Raw data is shown as open symbols (**⚬**) while curve fitted and envelope is represented by solid and dashed lines, respectively.

XPS spectra of in situ activated Au-Cu/CeO_2_ (I) and Au/CeO_2_ (II) over the Au 4*f* (A) and Cu 2*p*_3/2_ (B) binding energy (BE) regions are represented in Figure 4, and the results after deconvolution given in Table 1. The Au 4*f* profiles for Au-Cu/CeO_2_ and Au/CeO_2_ are equivalent suggesting same electronic properties for the gold phase with no measurable electron transfer in Au-Cu/CeO_2_ relative to Au/CeO_2_. The XPS spectrum presents two peaks with associated binding energies (BE) at 83.5 and 87.2 eV corresponding to 4*f*_7/2_ and 4*f*_5/2_ levels, respectively [55]. The BE values are lower than those reported for Au^0^ (Au 4*f*_7/2_ = 84.0 eV and Au 4*f*_5/2_ = 87.7 eV [55,56]), indicative of Au-support interactions that impact on Au electronic character [57]. A similar 0.4-1.0 eV downshift in BE for Au nanoparticles ≤6 nm on CeO_2_ was reported by Lai et al. [58] and attributed to electron transfer from the support. The presence of Au*^δ^*^−^ nanoparticles is consistent with the adsorption/activation of the -C=O functionality in HMF via π-back donation [32]. The XPS spectrum for Au-Cu/CeO_2_ over the Cu 2*p* region (Figure 4(IB)) is characterised by a Cu 2*p*_3/2_ contribution at BE = 928.7 eV, corresponding to Cu^0^ (35%) [59]. A signal at higher BE (= 932.0 eV) with greater intensity can be linked to the presence of Cu^+^ (65%) [59]. The surface composition and oxidation state of copper from XPS measurements is consistent with TPR results. The XPS spectra over the Ce 3*d* region for CeO_2_, Au-Cu/CeO_2_ and Au/CeO_2_ are presented in Figure S1, Supplementary Materials. The as-received and H_2_-treated (to 573 K) CeO_2_ samples show an equivalent response characterised by a Ce^3+^ atomic ratio = 0.12 ± 0.01, indicative of a higher temperature requirement for Ce^4+^ → Ce^3+^ reduction (>625 K) [42]. A similar higher Ce^3+^ atomic ratio (0.26 ± 0.03) was recorded for both supported catalysts (Table 1). The presence of Au on CeO_2_ surface can induce a partial reduction of the support, due to hydrogen spillover [42,43], in agreement with H_2_ consumption during TPR analysis (Table 1).

### 2.3. Dehydrogenation of Alcohols Coupled with Hydrogenation of meta-Substituted Furaldehydes

**Figure 5:**
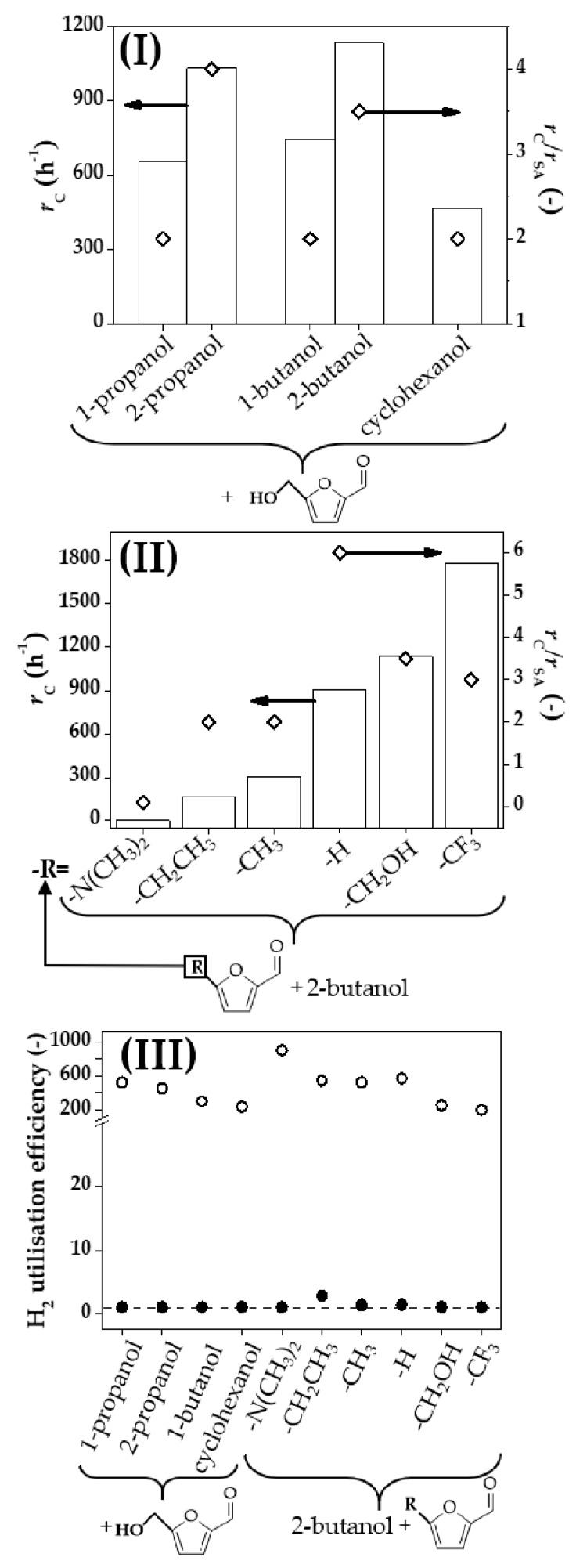
Comparison of hydrogenation performance over Au-Cu/CeO_2_ in the coupled process vs. stand-alone reaction using Au/CeO_2_: Hydrogenation rate in the coupling reaction (*r*_C_, bars) and rate enhancement (in the coupling vs. stand-alone hydrogenation given as *r*_C_/*r*_SA_, ◇) for the conversion of (**I**) a series of alcohols + HMF and (II) 2-butanol + *m*-substituted furaldehydes; (**III**) H_2_ utilisation efficiency in coupling system (●) and in stand-alone hydrogenation using an external H_2_ supply (**⚬**). *Note:* horizontal dashed line in (**III**) represents full H_2_ utilisation under stoichiometric conditions. *Reaction conditions*: *P* = 1 atm, *T* = 498 K.

We examined the potential of the Au-Cu/CeO_2_ catalyst for coupling a range of alcohols with *m*-substituted furaldehydes and the results are presented in Figure 5. In each case, we achieved full selectivity to the target aldehyde/ketone (from dehydrogenation) and alcohol (from hydrogenation) with no evidence of undesired hydrogenolysis, ring reduction, dehydration or dimerisation. We first evaluated the effect of changing the nature of the alcohol in the dehydrogenation step and the results for the coupled process with HMF are shown in Figure 5(I). In each case, the rate of DHMF production was up to a 4-fold greater than that recorded over Au/CeO_2_ using an external hydrogen supply. The modified hydrogenation activity for the different alcohols (cyclohexanol < 1-propanol < 1-butanol < 2-propanol < 2-butanol) demonstrates that hydrogen generation (from dehydrogenation) is rate limiting in the coupling system. The greater hydrogenation rate for secondary vs. primary alcohols (2-butanol vs. 1-butanol and 2-propanol vs. 1-propanol) observed is in line with reports in the literature [60,61] and consistent with reaction thermodynamics where hydrogen extraction from 2-butanol (to methyl elthyl ketone; reduction potential $\Delta H_{f}^{o}$ = 69 kJ·mol^−1^ [62]) is more favourable than 1-butanol conversion (to butyl aldehyde, $\Delta H_{f}^{o}$ = 80 kJ·mol^−1^) [62]) as well as in case of 2-propanol (formation of acetone, $\Delta H_{f}^{o}$ = 70 kJ·mol^−1^ [62]) and 1-propanol (formation of propanal, $\Delta H_{f}^{o}$ = 86 kJ·mol^−1^ [62]). Greater $\Delta H_{f}^{o}$ (77 kJ·mol^−1^ [62]) and increased steric hindrance [63] can account for the lower dehydrogenation rate of (cyclic) cyclohexanol (to cyclohexanone) relative to secondary aliphatic alcohol conversions. 

In the dehydrogenation of 2-butanol coupled with hydrogenation of substituted furaldehydes bearing -CH_3_, -CH_2_CH_3_, -CH_2_OH, -CF_3_, -N(CH_3_)_2_ and -H substituents in the *meta-*position, Au-Cu/CeO_2_ was again fully selective in generating 2-butanone and the corresponding alcohol product. The higher hydrogenation activity in the coupling process over Au-Cu/CeO_2_ relative to stand-alone hydrogenation using Au/CeO_2_ observed in the case of HMF extended to all the substituted furaldehydes (Figure 5(II)). The following activity sequence was established in the coupling process: 5-dimethylamino-2-furaldehyde < 5-ethyl-2-furaldehyde < 5-methyl-2-furaldehyde < furfural < HMF < 5-trifluoromethyl-2-furaldehyde. The hydrogenation of the carbonyl group has been proposed to occur through a nucleophilic mechanism [64] with formation of a negatively charged hydroxyalkyl intermediate [65] that results from the attack of the carbonyl oxygen by hydrogen that acts as a weak nucleophilic agent [66]. Electron withdrawing functionalities in *meta-*position of aromatic systems favour the delocalisation of the negative charge in the hydroxyalkyl intermediate lowering the activation energy barrier [65] of the first reaction step (i.e. addition of the first hydrogen), which, in turns increases hydrogenation rate. The *σ*_m_ factor (Hammet constant) is an empirical parameter that provides a measure of the electron donating/acceptor character of a functionality in *meta-*position on aromatic systems [67,68]. In a nucleophilic attack, reaction rate is increased by electron-withdrawing groups with σ_m_ > 0. The higher activity with increasing σ_m_ (from -N(CH_3_)_2_ σ_m =_ −0.16 to CF_3_ σ_m_ = 0.43) [63] (see Figure 5(II)) is consistent with a nucleophilic mechanism. 

The hydrogen utilisation efficiency (see Material and Methods) in the conversion of the different *m*-furaldehydes by stand-alone hydrogenation (using conventional pressurised H_2_) and coupling is presented in Figure 5(III). The hydrogen utilisation for stand-alone hydrogenation of all the tested furaldehydes was 200–900 times greater than the stoichiometric (=1) requirements, a result that represents high inefficiency. This issue was circumvented in the coupled process where, in each case, hydrogen utilisation efficiency was close or equal to stoichiometry with an associated E-factor 3–15 times lower relative to stand alone hydrogenation.

## 3. Materials and Methods 

### 3.1. Catalyst Preparation and Activation

The CeO_2_ support was purchased from Sigma-Aldrich and used as received. 1 mol% Au/CeO_2_ was prepared by deposition–precipitation using urea (Riedel-de Haën, 99%) as basification agent. An aqueous solution of urea (3 M) and HAuCl_4_·H_2_O (6 × 10^-4^ M, 300 cm^3^, Sigma-Aldrich, 99.995%) was added to the support (5 g) and the suspension was stirred (600 rpm) and heated to 353 K. The pH progressively increased to reach 7 as a result of thermal decomposition of urea with consequent formation of Au(III) surface complexes [69]. The (1 mol% Au and 2 mol% Cu) Au-Cu/CeO_2_ catalyst was prepared by stepwise deposition–precipitation of Cu followed by Au. NaOH (2 M, Fisher Scientific, ≥97%) as basification agent was added to an aqueous solution of the metal precursor (Cu(NO_3_)_2_·3H_2_O (1 × 10^−2^ M, 200 cm^3^ Sigma-Aldrich, 99%)) containing the support (5 g) until pH = 10, heated to 353 K and aged under vigorous stirring for 4 h to ensure homogeneous deposition of Cu(OH)_2_ [70]. Au incorporation to the Cu/CeO_2_ catalyst was performed by deposition–precipitation, method as above. The solid obtained was filtered, washed with distilled water until pH = 7 and dried at 393 K overnight. Prior to use in catalysis, the samples (sieved into a batch of 75 μm average diameter) were activated in 60 cm^3^ min^−1^ H_2_ at 2 K min^−1^ to 573 K, which was maintained for 1 h. The samples were cooled to ambient temperature and passivated in 1% *v/v* O_2_/N_2_ at 298 K for ex situ characterisation.

### 3.2. Catalyst Characterisation

The Au and Cu loading was measured by atomic absorption spectroscopy (AAS) using a Shimadzu AA-6650 spectrometer with an air-acetylene flame from the diluted extract in aqua regia (25% *v/v* HNO_3_/HCl). Temperature programmed reduction (TPR) was conducted on the CHEM-BET 3000 (Quantachrome Instrument) unit with data acquisition/manipulation using the TPR WinTM software. Samples were loaded into a U-shaped Pyrex glass cell (3.76 mm i.d.) and heated in 17 cm^3^ min^−1^ (Brooks mass flow controlled) 5% *v/v* H_2_/N_2_ to 573 K at 2 K min^−1^. The effluent gas passed through a liquid N_2_ trap and H_2_ consumption was monitored by a thermal conductivity detector (TCD). Curve fitting TPR profile (CasaXPS 2.3.17 software) was employed to analyse the reduction step(s) during thermal treatment [71]. Metal particle morphology (size and shape) was examined by STEM probe corrected on a JEOL ARM 200CF with an energy dispersive X-ray (EDX) detector operated at an accelerating voltage of 200 kV. The scanned images were collected using either Gatan 806 High Angle Annular Dark Field, Gatan 805 Annular Dark Field/Bright field or JEOL ADF1detectors under the control of a Gatan DigiScan II, employing Gatan DigitalMicrograph software (version 2.31) for data acquisition/manipulation. Samples were prepared for analysis by dry deposition on a holey carbon/Ni grid (300 Mesh). At least 250 individual metal nanoparticles were counted for each catalyst and the surface area-weighted mean metal particle size (*d*_STEM_) was calculated from:

(1) $$d_{STEM}=\frac{\sum_{i}n_{i}d_{i}^{3}}{\sum_{i}n_{i}d_{i}^{2}}$$

where *n_i_* is the number of particles of diameter *d*_i_. X-ray photoelectron spectroscopy (XPS) measurements were performed using a monochromatised Al anode (K_α_ 1486.6 eV, 10 kV, 20 mA). Prior to analysis, the samples were activated in situ in a pre-chamber under H_2_ atmosphere (10^2^ mbar) at 2 K min^−1^ to 573 K. The source power was maintained at 3.9 × 10^3^ W and the emitted photoelectrons were sampled from an area of 13 mm^2^; the photoelectron take-off angle was normal emission (0°). The analyser pass energy was 150 eV for survey (0–1100 eV) and high-resolution spectra (over the Au 4*f*, Cu 2*p* and Ce 3*d* core levels). The C 1*s* peak was calibrated at 284.5 eV and used as internal standard to compensate for charging effects. Spectra curve fitting and quantification were performed with the CasaXPS software, using relative sensitivity factors provided by Kratos.

### 3.3. Catalytic Procedure

Reactions (independent hydrogenation of *m-*furaldehyde in H_2_, alcohol dehydrogenation in N_2_ and coupled alcohol dehydrogenation/*m-*furaldehyde hydrogenation in N_2_) were carried out under atmospheric pressure, in situ immediately after activation, in a fixed bed vertical continuous flow glass reactor (*i.d.* = 15 mm) at 498 K. Operating conditions ensured negligible internal/external mass and heat transfer limitations [72]. A layer of borosilicate glass beads served as preheating zone where the organic reactant(s) was(were) vaporised and reached reaction temperature before contacting the catalyst bed. Isothermal conditions (± 1 K) were maintained by thoroughly mixing the catalyst with ground glass (75 µm). Reaction temperature was continuously monitored by a thermocouple inserted in a thermowell within the catalyst bed. The reactant(s) was(were) delivered to the reactor via a glass/Teflon air-tight syringe and Teflon line using a microprocessor-controlled infusion pump (Model 100 kd Scientific). Stand-alone hydrogenation and dehydrogenation were carried out in a co-current flow of H_2_ with *m-*furaldehyde (*GHSV* = 3.4 × 10^3^ h^− 1^; molar Au to reactant feed rate (*n*_Au_/*F_m_*_-furaldehyde_) = 1 × 10^− 3^ h) or N_2_ with alcohol (*GHSV* = 3.4 × 10^3^ h^−1^, *n*_Au_/*F*_alcohol_ = 3 × 10^−2^ h). The coupled reaction was carried out in N_2_ (*GHSV* = 3.4 × 10^3^ h^− 1^, *n*_Au_/*F_m_*_-furaldehyde_ = 2 × 10^− 4^ – 1 × 10^− 3^ h). The catalytic response over Au-Cu/CeO_2_ vs. Au/CeO_2_ in the stand-alone hydrogenation of HMF (using an external hydrogen supply) was examined by using an aprotic solvent (anisole) to avoid any contribution to hydrogenation from in situ hydrogen production via alcohol dehydrogenation over Cu. In a series of blank tests, passage of each reactant in a stream of H_2_ or N_2_ through the empty reactor or over the (CeO_2_) support alone did not result in any detectable conversion. The reactor effluent was condensed in a liquid nitrogen trap for subsequent analysis using a Perkin-Elmer Auto System XL gas chromatograph equipped with a programmed split/splitless injector and a flame ionisation detector, employing a DB-1 (50 m × 0.33 mm *i.d.*, 0.20 μm film thickness) capillary column (J&W Scientific). Data acquisition and manipulation were performed using the TurboChrom Workstation Version 6.3.2 (for Windows) chromatography data system. Furfural (Sigma-Aldrich, 99%), 5-methyl-2-furaldehyde (Sigma-Aldrich, >98%), 5-ethyl-2-furaldehyde (Sigma-Aldrich, 98%), 5-hydroxymethyl-2-furaldehyde HMF (Sigma-Aldrich, 99%), 5-trifluoromethyl-2-furaldehyde (Sigma-Aldrich), 5-dimethylamino-2-furaldehyde (Sigma-Aldrich), 1-butanol (Sigma-Aldrich, 99.8%), 2-butanol (Sigma-Aldrich, 99.5%) 1-propanol (Fisher Scientific, >99%), 2-propanol (Fisher Scientific, >99.5%), cyclohexanol (Fisher Scientific, >98%) and anisole (Sigma-Aldrich, 99.7%) were used as supplied without further purification. All gases (O_2_, H_2_, N_2_ and He) were of ultra-high purity (>99.99%, BOC). Reactant (i) fractional conversion (*X*_i_) is defined by:

(2) $$X_{i}=\frac{{\left[\mathrm{reactant}\right]}_{i,\mathrm{in}}-{\left[\mathrm{reactant}\right]}_{i,\mathrm{out}}}{{\left[\mathrm{reactant}\right]}_{i,\mathrm{in}}}$$

while selectivity to product *j* (*S*_j_) is defined as:

(3) $$S_{j}(\%)=\frac{{\left[\mathrm{product}\right]}_{j,\mathrm{out}}}{{\left[\mathrm{reactant}\right]}_{i,\mathrm{in}}-{\left[\mathrm{reactant}\right]}_{i,\mathrm{out}}}\times 100$$

where subscripts in and out refer to the inlet and outlet streams. The catalysts exhibited a decline in conversion to reach a pseudo-steady state after 2 h on-stream. Catalytic activity is also quantified in terms initial rate (*r*_C_ = hydrogenation in coupling process and *r*_SA _ = hydrogenation in stand-alone reaction; mol mol_Au_^−1^ h^−1^), determined from time on-stream measurements as described elsewhere [72] according to:

(4) $$r_{C\;\mathrm{or}\;\mathrm{SA}}=\frac{F_{m-\mathrm{furaldehyde}}\times X_{i}}{n_{\mathrm{Au}}}$$

Hydrogen utilisation efficiency in the stand-alone hydrogenation vs. coupled process was assessed by:

(5) $$H_{2}utilisation\;efficiency=\frac{H_{2}\mathrm{supply}}{H_{2}\mathrm{consumed}}$$

where *H*_2_ supply is the molar hydrogen provided ((i) from an external gas cylinder supply or (ii) via alcohol dehydrogenation) while *H*_2_ consumed is the amount utilised in the conversion of *m*-furaldehyde. Repeated reactions with different samples from the same batch of catalyst delivered raw data reproducibility and carbon mass balance within ± 5%.

## 4. Conclusions

Activation in hydrogen of Au-Cu/CeO_2_ (Cu/Au mol ratio = 2) prepared by stepwise deposition–precipitation generated metal nanoparticles in the range 1–7 nm (mean = 3.5 nm). STEM-EDX analysis of Au-Cu/CeO_2_ has revealed that Au and Cu nanoparticles are in close proximity on the surface while XPS results are consistent with formation of Au*^δ−^*, Cu^0^ and Cu^+^. Au/CeO_2_ (bearing Au*^δ−^*nanoparticles with mean size = 3 nm) promoted the gas phase continuous hydrogenation of HMF (*P* = 1 atm, *T* = 498 K) exclusively to DHMF with only a small fraction of the hydrogen supplied being utilised. Under the same reaction conditions, Au-Cu/CeO_2_ delivered a higher DHMF production rate and full hydrogen utilisation in the coupled hydrogenation/dehydrogenation of HMF/2-butanol with a lower E-factor. Exclusive carbonyl-group hydrogenation and hydroxyl-group dehydrogenation with (up to a 6-fold) increase hydrogenation rate and hydrogen utilisation efficiency with a lower E-factor (relative to conventional stand-alone hydrogenation) extends to the coupling of a series of *m-*substituted (-CH_3_, -CH_2_CH_3_, -CH_2_OH, -CF_3_, -N(CH_3_)_2_ and -H) furaldehydes with alcohol (1-propanol, 1-butanol, 2-propanol, 2-butanol, cyclohexanol) dehydrogenation over Au-Cu/CeO_2_. Hydrogen generation is rate limiting and furaldehyde hydrogenation proceeds via a nucleophilic mechanism where the presence of electron withdrawing substituents (in the *meta*-position) is shown to increase hydrogenation rate. Our results open new possibilities for the application of bimetallic Au-Cu catalysts for sustainable hydrogenation/dehydrogenation coupling directed at production of high value chemicals from furaldehydes.

## Figures and Tables

**Table 1:** Physico-chemical characteristics of CeO_2_ supported Au-Cu and Au catalysts.

			Au-Cu/CeO_2_	Au/CeO_2_
**Metal loading (mol%)**			1 (Au)/2 (Cu)	1
**TPR**	***T*_max_ (K)**	**α**	414	410
		**β**	429	423
		**γ**	437	-
		**δ**	443	-
	**H_2_ consumption (µmol g^−1^)**		340 ^a^/63 ^b^/61 ^c^	307 ^a^/69 ^b^
***d*_STEM_** **(nm) ^d^**			4	3
**XPS**	**Binding energies (eV)**	Au^0^ (%)	83.6 (100)	83.5 (100)
		Cu^0^ (%)	928.7 (35)	
		Cu^+^ (%)	932.0 (65)	
	**Ce^3+^ atomic ratio ^e^**		0.24	0.29

^a^ experimental value; ^b^ theoretical hydrogen requirement for Au^3+^ → Au^0^; ^c^ theoretical amount of hydrogen for Cu^2+^ → Cu^+^ → Cu^0^; ^d^ mean metal particle size from scanning transmission electron microscopy (STEM) analysis (Equation (1)); ^e^ Ce^3+^ atomic ratio = Ce^3+^/(Ce^4+^ + Ce^3+^) from XPS measurements (see Figure S1 in Supplementary Materials). *Note: α, β, γ and δ represent hydrogen consumption peaks during TPR associated with transition of Au^3+^ → Au^0^, Ce^4+^ → Ce^3+,^ Cu^2+^ → Cu^+^ and Cu^+^ → Cu^0^, respectively.*

## Supplementary Materials

The following are available online, Figure S1: XPS spectra over the Ce 3*d* region for (I) fresh CeO_2_, (II) CeO_2_ thermally treated in H_2_ to 573 K, (III) Au-Cu/CeO_2_ and (IV) Au/CeO_2_. *Note:* Raw data are shown as open symbols (**⚬**) while curve fitted and envelope is represented by solid and dashed lines.
