# Supplementary material for: Gas Phase Hydrogenation of Furaldehydes via Coupling with Alcohol Dehydrogenation over Ceria Supported Au-Cu
Source: Molecules. 2018 Nov 7;23(11):2905. doi: 10.3390/molecules23112905 (PMC6278317; doi:10.3390/molecules23112905)
Supplement: Supplementary file 1 [file molecules-23-02905-s001.pdf]

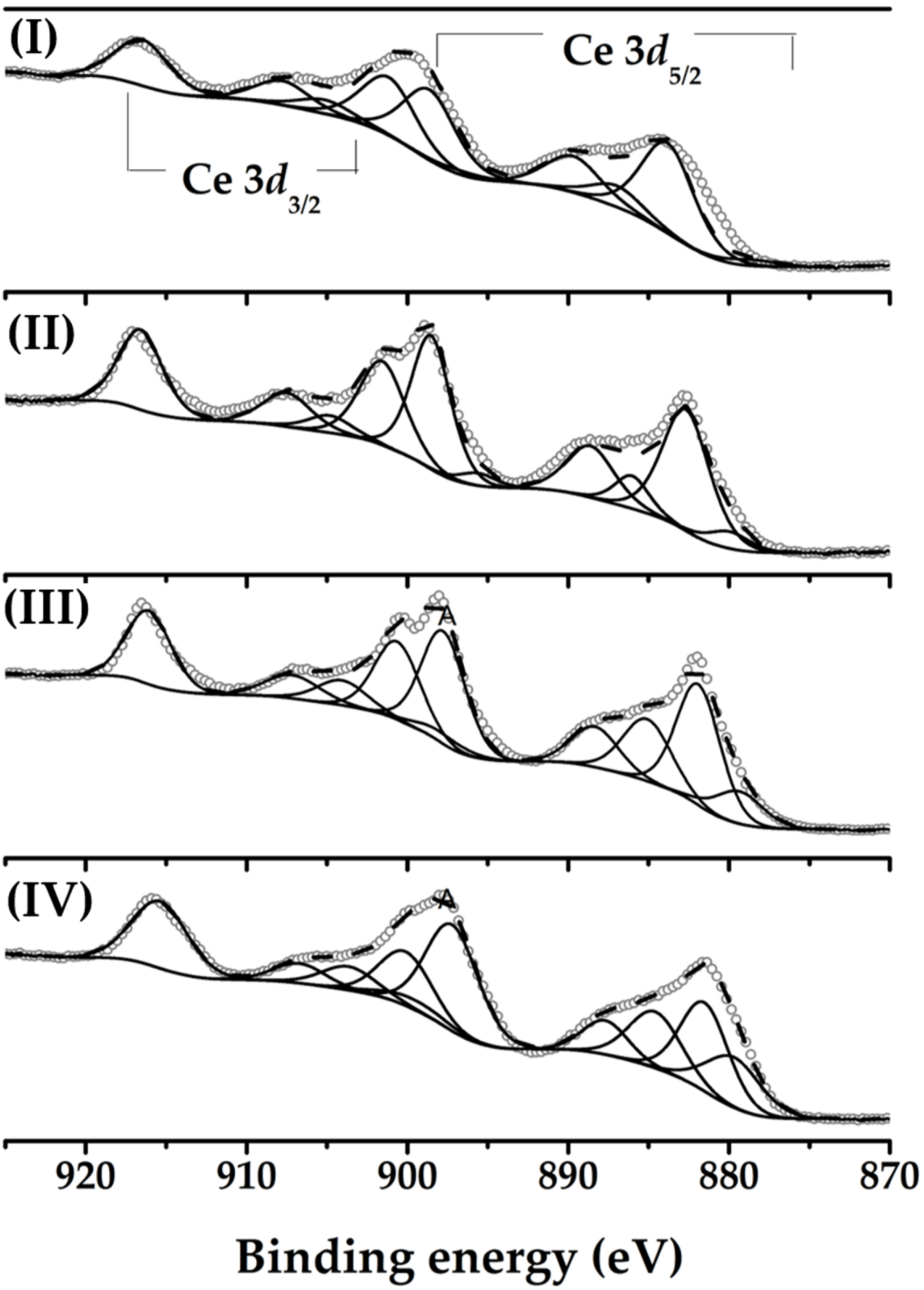


**Figure S1:** XPS spectra over the Ce 3*d* region for **(I)** fresh $CeO_2$, **(II)** $CeO_2$ thermally treated in $H_2$ to 573 K, **(III)** Au-Cu/$CeO_2$ and **(IV)** Au/$CeO_2$. *Note:* Raw data are shown as open symbols (○) while curve fitted and envelope is represented by solid and dashed lines.
